# X-ray Investigation of CsPbI_3_:EuCl_3_ Infiltrated into Gig-Lox TiO_2_ Spongy Layers for Perovskite Solar Cells Applications

**DOI:** 10.3390/nano13222910

**Published:** 2023-11-07

**Authors:** Paola La Magna, Carlo Spampinato, Salvatore Valastro, Emanuele Smecca, Valentina Arena, Giovanni Mannino, Ioannis Deretzis, Giuseppe Fisicaro, Corrado Bongiorno, Alessandra Alberti

**Affiliations:** 1 CNR-IMM, Zona Industriale Strada VIII n.5, 95121 Catania, Italy; paola.lamagna@imm.cnr.it (P.L.M.); carlo.spampinato@imm.cnr.it (C.S.); salvatore.valastro@imm.cnr.it (S.V.); emanuele.smecca@imm.cnr.it (E.S.); valentina.arena@imm.cnr.it (V.A.); giovanni.mannino@imm.cnr.it (G.M.); ioannis.deretzis@imm.cnr.it (I.D.); giuseppe.fisicaro@imm.cnr.it (G.F.); corrado.bongiorno@imm.cnr.it (C.B.); 2 Department of Mathematical and Computer Sciences, Physical Sciences and Earth Sciences, University of Messina, Viale F. Stagno d’Alcontres 31, 98166 Messina, Italy

**Keywords:** perovskite, CsPbI_3_, TiO_2_, XRD, lattice, texturing, europium, infiltration

## Abstract

In this study, we explore the potential of a blended material comprising ${\mathrm{CsPbI}}_{3}$:${\mathrm{EuCl}}_{3}$ perovskite and Gig-Lox ${\mathrm{TiO}}_{2}$, a unique transparent spongy material known for its multi-branched porous structure, for application in solar cells. The inclusion of ${\mathrm{EuCl}}_{3}$ in ${\mathrm{CsPbI}}_{3}$ serves to stabilize the photoactive γ-phase with a bandgap of 1.75 eV, making it suitable for solar energy conversion in tandem solar cells. Our study applies X-ray-based techniques to investigate the structural properties and interfacial behavior within this blended material, in comparison with a reference perovskite layer deposited on glass. In addition, Spectroscopic ellipsometry is complemented with density functional theory calculations and photoluminescence measurements to elucidate the absorption and radiative emission properties of the blend. Notably, our findings reveal a significant quenching of photoluminescence within the blended material, underscoring the pivotal role of the distributed interfaces in facilitating efficient carrier injection from the ${\mathrm{CsPbI}}_{3}$:${\mathrm{EuCl}}_{3}$ perovskite into the Gig-Lox ${\mathrm{TiO}}_{2}$ sponge. These findings pave the way for the application of the blend as an Electron Transport Layer (ETL) in semi-transparent perovskite solar cells for tandem and building integrated photovoltaics.

## 1. Introduction

Two related phenomena have emerged in recent decades: the quickening pace of industrialization and the rise in Earth’s surface temperature brought on by an increase in greenhouse gas concentrations in the atmosphere [1,2]. The scientific community was inspired to find a means to lower overall emissions by switching from numerous conventional energy sources to renewable ones like the sun, wind, and water because of the growing awareness of global warming. Bulk silicon is used as a photoactive component, which is the mainstay of the photovoltaic industry as it exists today. Though the usage of solar panels has spread widely over time, there is a risk that their production costs and energy consumption will prevent them from spreading far enough to meet Europe’s 2030 energy targets [3]. Perovskite presents as an alternative in this context, requiring less energy during manufacturing processes, cheaper components, and reduced production costs [4]. The National Renewable Energy Laboratory (NREL) chart shows that third-generation solar cells based on perovskite materials achieved an efficiency of 26.1% in a few years, the same value seen in silicon solar cells with exceptional performance [5]. In addition to the three-dimensional (3D) organic–inorganic hybrid perovskite solar cell (PSC) certified power conversion efficiency (PCE), the weak thermal stability of these devices, which is partly caused by the volatility of organic molecules, prevents their commercial implementation [6]. Inorganic cations, such as Cs, took the place of the volatile organic components to address this problem. Furthermore, ${\mathrm{CsPbI}}_{3}$ has a 1.75 eV optimum bandgap, making it suitable for tandem devices [7,8]. After 30 days of ageing, PCE of over 18% in ${\mathrm{CsPbI}}_{3}$ solar cells is claimed to be kept within 95% of the initial value [9]. Despite of having ideal characteristics, ${\mathrm{CsPbI}}_{3}$ experiences a spontaneous phase shift from a photo-active γ-phase (black phase) to a photo-inactive δ-phase (yellow phase) at ambient temperature [10,11]. Although it is currently difficult to achieve sufficiently extended stability in the ${\mathrm{CsPbI}}_{3}$ perovskite, it is well known that a variety of dopants can be employed to try to stabilise a black phase of ${\mathrm{CsPbI}}_{3}$, as reported in [12]. In our earlier research, we outlined several benefits of incorporating Eu into the perovskite formulation: (1) One can generate the photoactive γ-phase by annealing at 80 °C, a low temperature ([13,14,15]); (2) It is stabilised by a Eu-containing self-material edge at the grain boundaries, which creates a positive energy balance between volume costs and surface gain; (3) Eu contributes to a local charge compensation by taking up interstitial positions that support the robustness of the perovskite lattice. The integration of ${\mathrm{CsPbI}}_{3}$ onto silicon tandem solar cells can benefit from the usage of Eu. In the case of a monolithic tandem device, low-temperature procedures (<200 °C) are required to safeguard the bottom cell because the perovskite solar cell is deposited right on top of the silicon cell.

**Figure 1:**
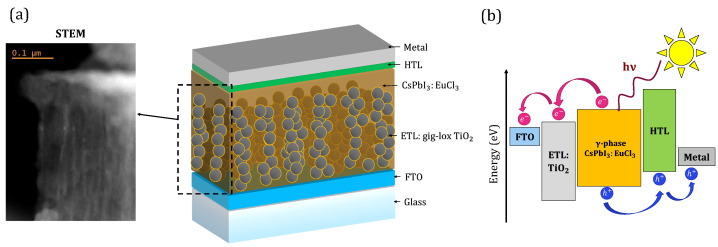
(**a**) Schematic figure of a typical structure of a perovskite solar cell coupled with a STEM image of the blended material made by ${\mathrm{CsPbI}}_{3}$:${\mathrm{EuCl}}_{3}$ and a Gig-Lox ${\mathrm{TiO}}_{2}$ layer. (**b**) Working principle of the perovskite solar cell device integration of the blends.

The critical function of the photoactive layer in a solar cell’s structure is the transformation of incoming photons into electron–hole pairs. However, electron (ETL) and hole (HTL) transporting layers must be included in the architecture to prevent charge recombination, which can reduce the device’s efficiency [16]. ${\mathrm{TiO}}_{2}$’s high electron mobility and strategic energy levels make it a viable choice for use as an electron collector in ${\mathrm{CsPbI}}_{3}$-based devices according to literature [17]. Spongy ${\mathrm{TiO}}_{2}$ layers are typically created chemically and incorporated into cell architecture to improve electron extraction [18,19]. As an alternative, physical growth techniques without solvents can be scaled up for large manufacturing volumes [20,21]. The Gig-Lox ${\mathrm{TiO}}_{2}$ layer is one of the newer ones that can be used in PSCs as 1D structures with [001] favoured orientation [22]. Its branching porosity allows for versatile usage in Pb capture in damaged PSC [23,24]. The first integration of ${\mathrm{CsPbI}}_{3}$ (with ${\mathrm{EuI}}_{2}$ addition) in Gig-Lox ${\mathrm{TiO}}_{2}$ layers can be found in ref. [25]. A solar cell device with a blended material between an active material infiltrated with ${\mathrm{CsPbI}}_{3}$:${\mathrm{EuCl}}_{3}$ perovskite and a Gig-Lox ${\mathrm{TiO}}_{2}$ layer is shown in Figure 1a. The perovskite into Gig-Lox ${\mathrm{TiO}}_{2}$ is responsible for the bright signal seen in the Scanning Transmission Electron Microscopy (STEM) image [26]. The PSC device’s operating principle is shown in Figure 1b. The creation of a blended material ensures that photo-generated electrons can flow and be extracted from a layered structure across an extended interface as opposed to a flat one.

However, as a result of the two materials blending together, deposition and infiltration of perovskite into a mesoporous substrate, like Gig-Lox ${\mathrm{TiO}}_{2}$, may cause a local structural rearrangement of the ${\mathrm{CsPbI}}_{3}$ crystals as well as a modification of the electronic properties that may have an impact on a photovoltaic cell’s performance [27].

This study indeed focuses on a widespread characterization of ${\mathrm{CsPbI}}_{3}$:${\mathrm{EuCl}}_{3}$ lattice structure and parameters after infiltration into a 340 nm-thick Gig-Lox ${\mathrm{TiO}}_{2}$ layer with 49% of porosity. We complemented the structural findings with the analysis of optical absorption and photoluminescence of the blended material. As a reference, we used a ${\mathrm{CsPbI}}_{3}$:${\mathrm{EuCl}}_{3}$ layer, 140 nm-thick, deposited on a bare glass substrate.

## 2. Materials and Methods

### 2.1. Gig-Lox TiO${}_{2}$ Deposition

Porous ${\mathrm{TiO}}_{2}$ can be grown through chemical or physical methods. While the chemical approaches are the most diffused and versatile ways to generate nano-${\mathrm{TiO}}_{2}$ architectures, the physical growth methods, like planar (parallel plate) sputtering deposition, are used to form compact layers. However, Sanzaro et al. [22] developed an innovative sputtering deposition method called Gig-Lox (Grazing Incidence Geometry assisted by Local OXidation) to grow ${\mathrm{TiO}}_{2}$ layers with a double-range porosity during an in situ growth process: a nano-porosity (1–5 nm) as Thornton’s model effect, and a mesoporosity (5–50 nm) arising from the Ti source that is at a 12.7° off-axis. The deposition is carried out at room temperature in an $O_{2}$ reactive atmosphere at a flow rate of 2 sccm by applying 140 W of power, 475 mA of current, and 295 V of voltage (power loading 6.9 W/cm^2^). The resulting growth rate is 4 nm/min. To increase the layer homogeneity throughout the sample surface, the anode–cathode spacing is fixed at 1.2 cm, and the substrate is under continuous rotation at 20 rpm. The method can be, in principle, applied to other metal oxides.

### 2.2. Eu-Doped CsPbI${}_{3}$ Deposition

To prepare a perovskite solution, we combined 1 M ${\mathrm{PbI}}_{2}$ and 1 M CsI from Tokyo Chemical Industry in a mixed solvent of DMF and DMSO in a 3:1 volume-to-volume ratio. We also prepared a solution of ${\mathrm{EuCl}}_{3}$ (99.99% pure) coating from Sigma-Aldrich (Taufkirchen, Germany) at a concentration of 0.1 M using the same mixed solvent. The solutions were stirred at room temperature for 1 h. Then, 1 mL of the ${\mathrm{PbI}}_{2}/\mathrm{CsI}$ (${\mathrm{PbI}}_{2}$ Tokyo chemical 99.99% pure, CsI Tokyo chemical > 99% pure) solution was mixed with 0.5 mL of the ${\mathrm{EuCl}}_{3}$ solution to achieve the desired stoichiometry for ${\mathrm{CsPbI}}_{3}$:${\mathrm{EuCl}}_{3}$ samples and then stirred for 1 h. Throughout the entire process, the ambient air had a relative humidity of approximately 35%.

**Figure 2:**
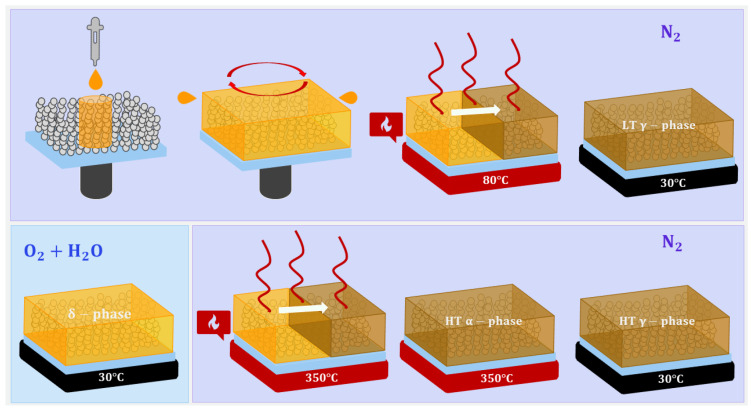
Representation of the deposition method of ${\mathrm{CsPbI}}_{3}$:${\mathrm{EuCl}}_{3}$ by spin-coating into Gig-Lox ${\mathrm{TiO}}_{2}$.

The deposition process of the perovskite on Gig-Lox ${\mathrm{TiO}}_{2}$ or a glass substrate is reported in the protocol shown in Figure 2. The procedure consists of a spin coating step in dry-$N_{2}$ atmosphere (RT ≈ 25 °C and humidity ≤ 10%). After the annealing (200 °C for 30 min) of the substrate to clean the surface from the potential presence of water and to increase the wettability [28], the ${\mathrm{CsPbI}}_{3}$:${\mathrm{EuCl}}_{3}$ stoichiometric solution was spin-coated on it at 1000 rpm for 10 s; after that, the rotational speed was increased to 3500 rpm for 25 s. Subsequently, the sample was placed on a heating stage at 80 °C for 10 min to let the solution evaporate and reach a black colour; to avoid further evolution from the black phase to the non-perovskite yellow one, a rapid quenching from 80 °C to 30 °C of the sample is necessary. After the exposure to humid air, the sample was annealed at 350 °C. At the end of this procedure, we obtained a layer made of ${\mathrm{CsPbI}}_{3}$:${\mathrm{EuCl}}_{3}$ and anatase Gig-Lox ${\mathrm{TiO}}_{2}$, which we call the *blended material*.

### 2.3. Blended Material Characterization

#### 2.3.1. Spectroscopic Ellipsometry Analysis

Spectroscopic ellipsometry (SE) data were collected using a J.A. Woollam VASE instrument (J.A. Woollam Co., Lincoln, NE, USA). Data were collected at different angles below and above the Brewster angle of the glass substrate, over a wide range of wavelengths, 200–1240 nm (1–6 eV), with a step of 10 nm. Samples were analysed in a $N_{2}$-filled cell to prevent degradation.

#### 2.3.2. X-ray Diffraction and X-ray Reflection Analysis

The lattice structure was investigated by X-ray Diffraction (XRD). The measurements were performed using a D8-Discoved diffractometer (Bruker AXS GmbH, Karlsruhe, Germany) equipped with a high-precision goniometer (0.0001 Å), a Cu − Kα (λ = 1.54059 Å) source with an instrumental broadening of 0.07°, eventually soller slits at the primary beam, and with variable slits and a detector at the secondary path. An Anton Paar heating stage, equipped with a polyether ether ketone dome filled with dry $N_{2}$ at a pressure slightly above the atmospheric one (+0.3 bar), was used to keep the samples at a controlled temperature (30 °C). The XRD patterns were acquired with a step size of 0.02° with an acquisition time of 10 s per step in the 2Θ range of 13°–39°. In the X-ray Reflectivity analyses (XRR), the angular range used extended from zero towards the critical angle for total reflection (2$\Theta_{c}$ = 0.57° for ${\mathrm{CsPbI}}_{3}$:${\mathrm{EuCl}}_{3}$) and a little further beyond, with a step size of 0.002° and a time per step of 2 s. Above that, Kiessig Fringes were not observed due to the overall layer thickness and roughness.

#### 2.3.3. Photoluminescence Spectroscopy Analysis

Photoluminescence Spectroscopy (PL) measurements were performed using a commercial instrument (Arkeo, Cicci Research s.r.l., Grosseto, Italy). The samples were excited by a green laser (532 nm) at 45° of incidence (spot diameter of 1 mm). After waiting for an integration time of 1 s, the PL signal was acquired by a spectrometer.

## 3. Results and Discussion

### 3.1. X-ray Reflection

**Figure 3:**
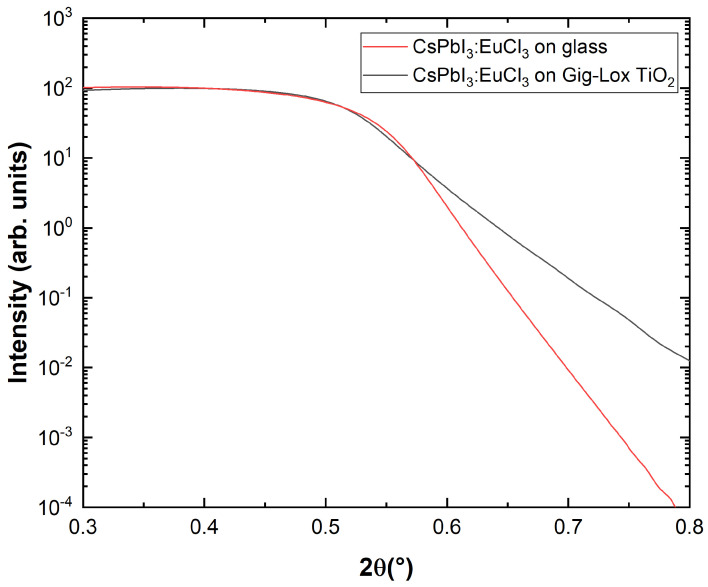
XRR fitted data of perovskite deposited on glass and on Gig-Lox ${\mathrm{TiO}}_{2}$.

XRR consists of collecting X-ray reflectivity curves at grazing incidents and allows determining parameters including thickness, density, and surface or interface roughness. We use this technique to measure the critical angle for total external reflection, which is linked to the density of the layer surface. The density provides the uppermost composition and is used to determine the penetration depth of X-rays inside the sample. The results shown in Figure 3 compare the data of the blended material with those of the reference case on bare glass.

The two samples present different roughness but similar critical angles $\Theta_{c}=0.285$°. We calculated the film density $\rho_{m}$ from the critical angle through the refractive index equation for X-rays [29]:

(1) n=1−δ−iβ,

where δ is the real parameter which depends on the X-ray wavelength, the film density and the composition of the material, and it is related to the critical angle according to the relation $\delta =\Theta_{c}^{2}/2$. The imaginary parameter β is associated with the linear absorption coefficient μ and the incident X-ray wavelength λ by equation β=λμ/4π.

Knowledge of critical angle $\Theta_{c}$ allows calculating the electron density $\rho_{\mathrm{el}}$ using the relation [29]:

(2) $$\rho_{\mathrm{el}}=\frac{2\pi \cdot \delta }{r_{e}\cdot \lambda^{2}}=\frac{\pi \cdot \Theta_{c}^{2}}{r_{e}\cdot \lambda^{2}}$$

where $r_{e}$ is the classical radius of an electron ($2.818\cdot 10^{-9}\;m$). Since the two samples have the same experimental $\Theta_{c}$, meaning the same electronic density at the sample surface, we argue that the blended material has a cap layer of pure perovskite over the filled Gig-Lox ${\mathrm{TiO}}_{2}$. Once calculating the electron density, the mass density $\rho_{m}$ is derived through function [29]:

(3) $$\rho_{m}=\frac{\rho_{\mathrm{el}}\cdot \left(A_{\mathrm{Cs}}+A_{\mathrm{Pb}}+3A_{I}\right)}{N_{A}\cdot \left(Z_{\mathrm{Cs}}+Z_{Pb}+3{\mathrm{ZI}}_{I}\right)},$$

where $N_{A}$ is the Avogadro number, $Z_{x}$ is the atomic number of the xth atom and $A_{x}$ is the atomic weight of the xth atom, each one multiplied by the atomic ratio (molar ratio) of the xth atom. The calculated value of $\rho_{m}$ is $4.707\;g/cm^{3}$ in both cases, while they differ for the roughness that is higher in ${\mathrm{CsPbI}}_{3}$:${\mathrm{EuCl}}_{3}$ on the glass substrate.

### 3.2. X-ray Diffraction

Structural insights on the blended material were derived from XRD data which provides essential information, such as texturing coefficients, crystallite sizes and lattice parameters. In order to explore the properties of ${\mathrm{CsPbI}}_{3}$:${\mathrm{EuCl}}_{3}$ as a cap layer and as infiltrated material, we collected data in the grazing incidence angle (GIXRD) and symmetric 2Θ/ω configurations.

#### 3.2.1. Grazing Incidence XRD

In the GIXRD setup, the incidence angle ω is fixed to small angles of 0.2°, 0.3°, 0.4°, 0.6° and 0.8°, starting close to the critical value where total reflection occurs, while the detector moves on the 2Θ circle to collect the signal. Associated with the incidence angle, we provide an evaluation of the probed thickness by calculating the penetration depth of X-rays.

Conventionally, the penetration depth is defined as the depth where the intensity of X-rays is reduced to 1/e (about 37%) of its intensity at the surface. The penetration depth of X-rays, **z**, is calculated starting from the imaginary parameter β of the refractive index **n** (see Equation (1)). The absorption coefficients μ of ${\mathrm{CsPbI}}_{3}$ compounds are obtained according to simple additivity of the elemental mass attenuation coefficients, ${\left(\mu /\rho \right)}_{i}$ [30]:

(4) $$\mu =\rho_{m}\sum_{i}\frac{M_{i}}{M}\cdot {\left(\frac{\mu }{\rho }\right)}_{i},$$

where $M_{i}$ is the mass fraction of the certain element in the ${\mathrm{CsPbI}}_{3}$ compound and M is its full mass. The values for the elemental mass attenuation coefficients, ${\left(\mu /\rho \right)}_{i}$, of Cs, Pb and I for the $Cu-K_{\alpha }$ radiation energy, are taken from ref. [31]. In GIXRD, the penetration depth depends on the electronic density $\rho_{m}$ of the probed material. This is different in the pure perovskite ($\rho_{m}=4.707\;g/cm^{3}$) with respect to the blend of ${\mathrm{TiO}}_{2}$ and perovskite ($\rho_{m}=4.298\;g/cm^{3}$) once the grazing incidence angle is fixed. The electronic density is indeed measured in the two cases by XRR and combined with GIXRD analyses.

**Figure 4:**
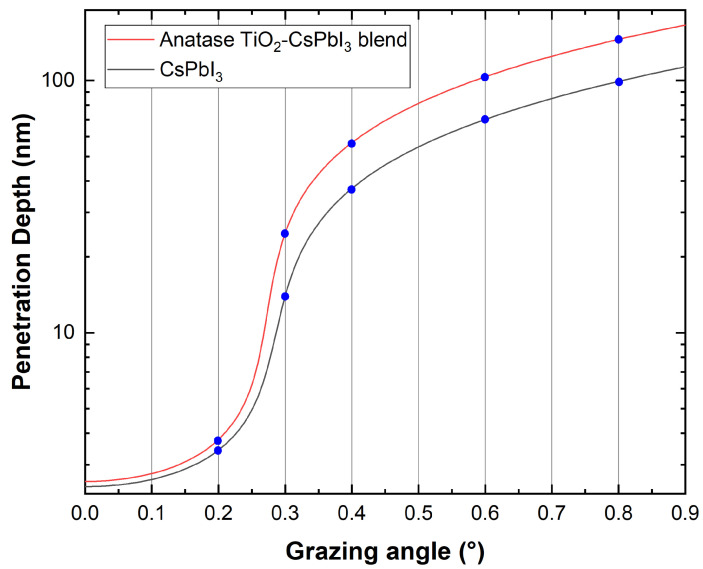
X-ray penetration depth with an incidence angle in the range of Θ < 0.9°.

Using the previous relations, we calculate the theoretical penetration depth $z_{1/e}$ according to equation [32]

(5) $$z_{1/e}=\lambda /4\pi B,$$

where

(6) $$B=\frac{1}{\sqrt{2}}{\left\{{\left[{\left(\alpha_{i}^{2}-\alpha_{c}^{2}\right)}^{2}+4\beta^{2}\right]}^{1/2}-\left(\alpha_{i}^{2}-\alpha_{c}^{2}\right)\right\}}^{1/2},$$

with $\alpha_{i}$ being the incident X-ray angle and $\alpha_{c}$ being the critical angle obtained by XRR data. In Figure 4 and Table 1, we show the penetration depth at the corresponding incident X-ray angle in the range of interest and in the two cases.

**Figure 5:**
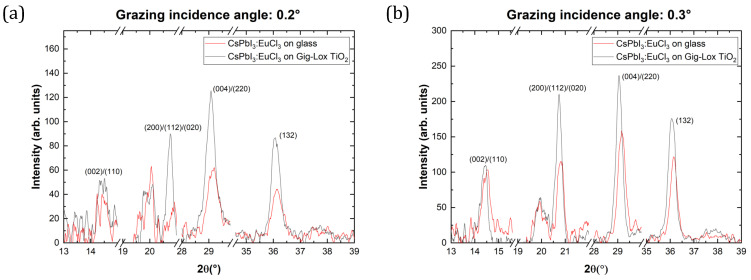

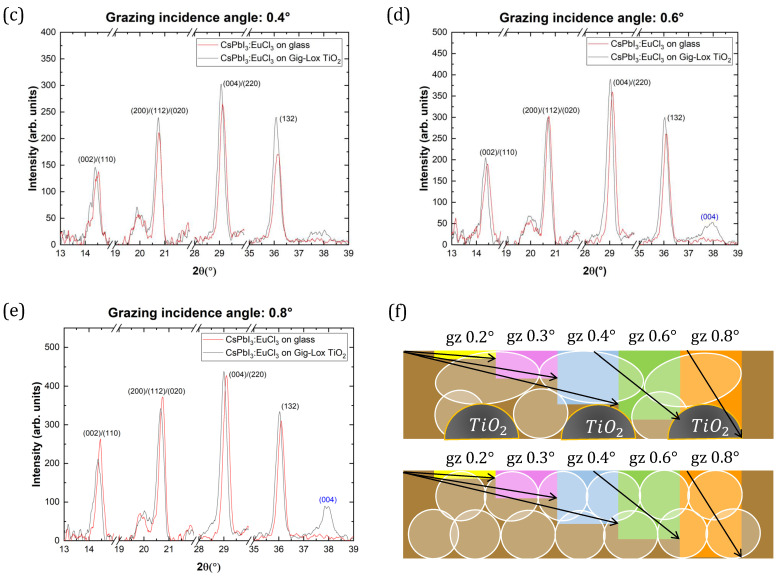
${\mathrm{CsPbI}}_{3}$:${\mathrm{EuCl}}_{3}$ single-layer and blended material GIXRD patterns at different fixed angles: 0.2° (**a**), 0.3° (**b**), 0.4° (**c**), 0.6° (**d**) and 0.8° (**e**). **(hkl)** are the ${\mathrm{CsPbI}}_{3}$:${\mathrm{EuCl}}_{3}$ crystallographic planes, (hkl) are the anatase-Gig-Lox ${\mathrm{TiO}}_{2}$ crystallographic planes. (**f**) Schematic representation of top layer crystallite sizes with different grazing angles (gz) on Gig-Lox ${\mathrm{TiO}}_{2}$ (top) and on bare glass (bottom).

Figure 5 compares the XRD patterns acquired at the grazing angles of Figure 4. Full integrability of ${\mathrm{CsPbI}}_{3}$:${\mathrm{EuCl}}_{3}$ with Gig-Lox ${\mathrm{TiO}}_{2}$ is testified by the presence of the same crystallographic planes compared to the case on the glass substrate, without reporting any significant phase changes during the fabrication process. In the grazing angle scan taken at the incidence angle of 0.6° (Figure 5d), a diffraction peak at 2Θ=37.9° associated with the crystallographic planes (004) of anatase-${\mathrm{TiO}}_{2}$ starts contributing definitely above the noise, while it is not found at lower grazing angles. On the basis of the calculated penetration depth, we argue that the cap-thin layer of pure perovskite has a thickness in the range of 40nm<z<70nm. In addition, the intensity of the peaks changes depending on the grazing angle and different substrates: diffraction peaks of perovskite as a cap layer on Gig-Lox ${\mathrm{TiO}}_{2}$ present greater intensity compared to what occurs on the glass substrate using incidence angles of 0.2° and 0.3° (Figure 5a,b). We offer our interpretation of this trend considering the different Full Width at Half Maximum (FWHM), and, according to the Debye–Scherrer relation [33], we calculate the different crystallite sizes of the peak at 2Θ=20.7° as a good example. From the results listed in Table 2, the cap layer has bigger crystallite sizes than the perovskite deposited on the glass substrate. A schematic representation is reported in Figure 5f. Going to greater scan angles (≥0.6°), having passed the cap layer, XRD peaks tend to reach the same intensities (Figure 5c–e).

#### 3.2.2. 2Θ/ω Scan

Using the symmetrical 2Θ/ω configuration, hereinafter we analyse the characteristics of the perovskite material infiltrated along the entire thickness of the Gig-Lox ${\mathrm{TiO}}_{2}$. Through the collected data, we calculate and compare crystallographic factors such as crystallite size, texturing coefficient and lattice parameters.

**Figure 6:**
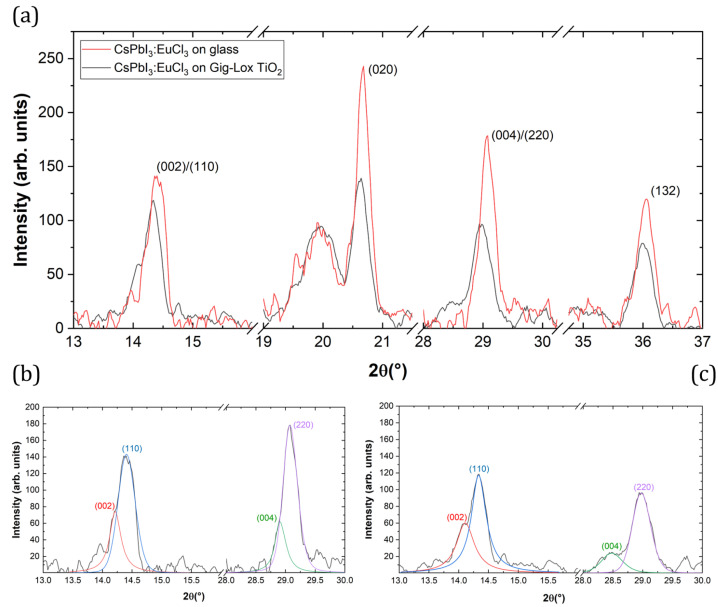
(**a**) Selected XRD patterns. Typical perovskite peaks appear at 14.4°, 20.6°, 28.9° and 36°. The peak at 2Θ = 19.9° is due to the instrumental setup. (**b**) Deconvolution peaks at 14.4° and 28.9° of ${\mathrm{CsPbI}}_{3}$:${\mathrm{EuCl}}_{3}$ on glass. (**c**) Deconvolution of the peaks at 14.4° and 28.9° of ${\mathrm{CsPbI}}_{3}$:${\mathrm{EuCl}}_{3}$ on Gig-Lox ${\mathrm{TiO}}_{2}$.

**Figure 7:**
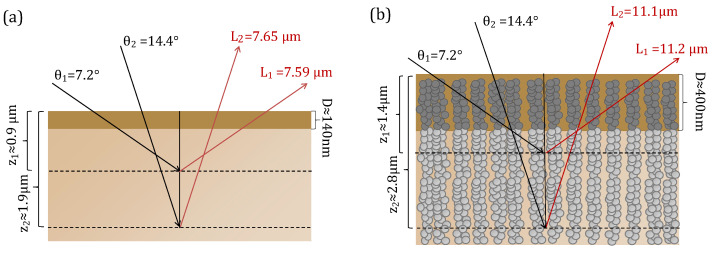
Different X-rays paths of bulk perovskite (**a**) and bulk blended material (**b**) at $\Theta_{1}=7.2$° and $\Theta_{2}=14.4$° with their output lengths $L_{1}$ and $L_{2}$.

**Figure 8:**
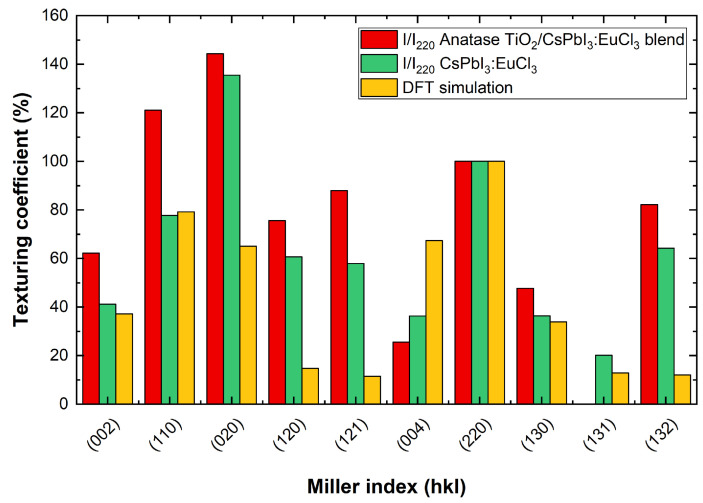
Texturing coefficient with different substrates compared to DFT calculation relative intensity.

In Figure 6, scans of the reference layer and the blended material are shown. Interplanar distance (d), crystallite size and texturing coefficient values using glass or Gig-Lox ${\mathrm{TiO}}_{2}$ or glass as substrate are listed in Table 3. The peaks of the perovskite layer infiltrated into the Gig-Lox scaffold are all wider than those of the same material grown on bare glass. We explain these results considering the different densities of the materials and therefore the different X-ray penetration depths. The schematic in Figure 7 depicts the different X-ray penetration paths **z** if we consider a bulk material of pure perovskite or that of blended material, while the effective thickness **D** that we probe in the prepared layers is highlighted depending on their real thickness. Different penetration depths at different angles also imply a different output length **L**. In the case of pure perovskite thin film (Figure 7a), when X-rays penetrate in the substrate with an incidence angle of $\Theta_{1}=7.2$°, an amount of 8.6% $L_{1}$ (output length) pass through **D**. Increasing the incidence angle to $\Theta_{2}=14.4$°, an amount of 4.6% $L_{2}$ (output length) passes through **D**, with the consequence of a greater absorption in the first case than in the second one. In this calculation, in addition to what is expected by Density Functional Theory (DFT) calculations (see details in ref. [11]) and the related expected scattering factors, the peak intensity at $2\Theta_{1}=14.4$° related to the (110) planes is further depleted compared to what measured at $2\Theta_{2}=28.9$° related to the (220) planes of the same family of Miller indexes. In the case of the blended material reported in Figure 7b, although 28.7% of $L_{1}$ and 14.3% of $L_{2}$ passes through **D**, we observe a more intense peak at $2\Theta_{1}$ than at $2\Theta_{2}$. This countertrend is under investigation. Besides this, we notice in the intensity values listed in Table 3 that the pure perovskite layer systematically has more intense peaks with respect to the blended material due to the different thicknesses of the films. A point-by-point comparison is provided in Figure 8 by the texturing coefficient defined as the relative intensity of each crystallographic plane with respect to the (220) plane. We show the values for the peaks associated with each crystallographic plane and the reference DFT [11] values normalised to what is measured or expected for the (220) crystallographic planes. We thus find a preferential growth of the perovskite crystals along the (020) planes for perovskites on glass and for the blended material with respect to what is expected by DFT.

Further information that we extract from diffraction data is the sizes of crystallites. From the FWHM values (Table 3), we obtain in the reference case the average size value of $Cry.Size_{av}=31.5\pm 8.9\;nm$, in the blended material case $Cry.Size_{av}=26.6\pm 2.8\;nm$. This behaviour is explained by the fact that crystallites on the glass substrate are free to assume their convenient size, whereas the structural confinement promotes the formation of crystallites with smaller dimensions inside the Gig-Lox ${\mathrm{TiO}}_{2}$.

From the values listed in Table 3, we observe a shift of the perovskite’s peak positions in the blended material with respect to the glass substrate. According to the Bragg relation, 2d·senΘ=nλ [34], this suggests a different interplanar space, and thus a different value of the three lattice parameters a, b and c [35]. To evaluate these new values, Rietveld refinement is used and the obtained results are listed in Table 4 compared to the literature values by Sutton et al. [36]. Lattice constants **a** and **c** expand on Gig-Lox ${\mathrm{TiO}}_{2}$ substrates with respect to the value obtained on the glass substrate, while **b** maintains similar values, resulting in an overall expansion of the volume of the unit cell. The results obtained are consistent with the porous nature of the ${\mathrm{TiO}}_{2}$ substrate with the fine pores allowing an inner adaptation of the intercalated perovskite, which is indeed free to expand with respect to a constrained compact film. The effects of lattice relaxation are also evident in Figure 6 with details of convoluted peaks at 14.4° and 28.9° displayed in Figure 6b,c, wherein perovskite’s (00l) family planes are more relevant in the blended material than the thin film on the glass.

### 3.3. Spectroscopic Ellipsometry

Identification of lattice distortion of crystals is important to properly study on the electronic properties of perovskite materials [37,38]. In the previous subsection, we calculated the volume of the unit cell in perovskite crystallites and we observed different lattice parameters and unit cell volume in the blended material with respect to the reference case. We complemented those findings with typos. The absorption coefficient of ${\mathrm{CsPbI}}_{3}$:${\mathrm{EuCl}}_{3}$ was obtained by ellipsometric measurements in the two cases.

**Figure 9:**
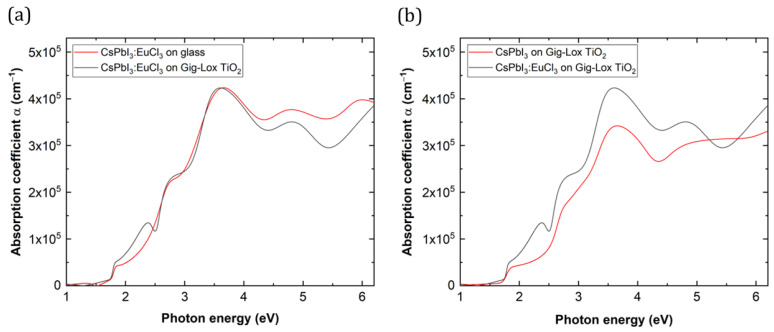
Absorption coefficient at different energies of (**a**) ${\mathrm{CsPbI}}_{3}$:${\mathrm{EuCl}}_{3}$ on glass and on Gig-Lox ${\mathrm{TiO}}_{2}$ (**b**) ${\mathrm{CsPbI}}_{3}$:${\mathrm{EuCl}}_{3}$ and ${\mathrm{CsPbI}}_{3}$ on Gig-Lox ${\mathrm{TiO}}_{2}$.

Figure 9a shows that the absorption spectra of perovskite films are similar in terms of bandgap energy value (1.75 eV) and absorption peaks at higher energies. We observed an extra absorption contribution at ≈2.4 eV on the blended material associated with the interaction of Eu with the ${\mathrm{TiO}}_{2}$ surfaces. This feature is not found in similar blended material without Eu in the perovskite (Figure 9b). A good compatibility of ${\mathrm{CsPbI}}_{3}$:${\mathrm{EuCl}}_{3}$ with the ${\mathrm{TiO}}_{2}$ substrate was indeed addressed. Despite the different lattice parameters and unit cell volume, the behaviour of the two materials in absorbing photons is similar or even slightly better in the blend material within the visible range.

### 3.4. Photoluminescence Spectroscopy

**Figure 10:**
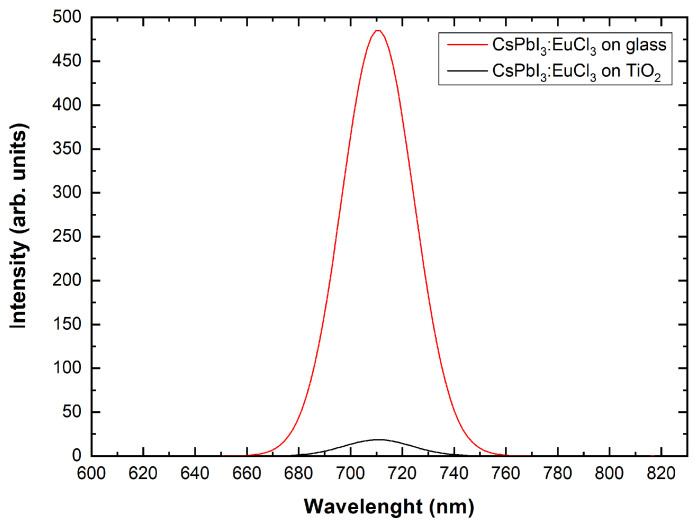
PL spectra at different wavelengths of ${\mathrm{CsPbI}}_{3}$:${\mathrm{EuCl}}_{3}$ on glass and on Gig-Lox ${\mathrm{TiO}}_{2}$.

To explore the applicability of the blended material on solar cell devices, it is fundamental to understand the role played by ${\mathrm{TiO}}_{2}$ in charge extraction. For this purpose, we perform photoluminescence (PL) measurements under a $N_{2}$ environment. Figure 10 displays the emission spectra of the blended material and the reference perovskite/glass sample: notably, the blended material exhibits a drastically lower PL intensity compared to the perovskite/glass case, with an area quenching of 94%.

We notice that, in both cases, the PL peak is centred at ≈710 nm, corresponding to a bandgap of 1.75 eV. In short, the blended material and the reference perovskite layer exhibit the same behaviour in absorption (Figure 9a) and photoemission (Figure 10), without any significant effect related to the slightly different unit cell volume [37,39]. The combined findings thus address the occurrence of an effective charge carrier injection from the infiltrated perovskite into the Gig-Lox ${\mathrm{TiO}}_{2}$ compared to what occurs in the reference layer perovskite on glass. These findings emphasise the pivotal role of the distributed interfaces for the carrier exchange into the blend. The expected technological impact of using the blended material as ETL is on the short circuit current of the device.

## 4. Conclusions

Using systematic comparisons to the reference case of perovskite layers formed on bare glass substrates, we examined the optical behaviour and the crystal structure of ${\mathrm{CsPbI}}_{3}$:${\mathrm{EuCl}}_{3}$ infiltrated into Gig-Lox ${\mathrm{TiO}}_{2}$ in this work. The unique properties of Gig-Lox ${\mathrm{TiO}}_{2}$, such as its ability to retain porosity during annealing, its multi-branched structure that facilitates good perovskite solution infiltration during deposition, and its appropriate energy levels as ETL, are what drive its adoption.

Understanding the behaviour of this system once it is infiltrated into the Gig-Lox ${\mathrm{TiO}}_{2}$ with a specified thickness is crucial for addressing future uses of this system for optoelectronic devices. In order to achieved this, we concentrated our crystallographic investigation on the 340 nm thick blended material’s interior as well as its interfaces, which can be used in solar cells. We identified the highest portion of the system using the XRR and GIXRD approaches, and we saw that the blended material had larger crystallites than the reference sample and that a pure perovskite cap layer was present over it. Through symmetrical 2Θ/ω XRD, the interior portion of the blended material was examined. We discovered a systematic lattice relaxation of ${\mathrm{CsPbI}}_{3}$:${\mathrm{EuCl}}_{3}$ crystals infiltrated into Gig-Lox ${\mathrm{TiO}}_{2}$.

We evaluated the absorption capability of the blended material by measuring the absorption coefficient using spectroscopic ellipsometry. Despite the slightly different lattice parameters of the blended material with respect to the reference, we noticed comparable features, except for a contribution ascribed to Eu interaction with the ${\mathrm{TiO}}_{2}$ surfaces. According to a similar behaviour under absorption with comparable bandgap energy, photoemission by the blended material is centred at the same value as in the reference perovskite on glass. As a noteworthy difference, a decrease in the PL intensity by 94% is observed in the blend which testifies to an effective injection of photo-generated carriers from the perovskite into the ${\mathrm{TiO}}_{2}$ through the many established interfaces.

All the experiments presented in this work were conducted under nitrogen environment that, once preserving the perovskite from degradation, allowed exploring the integrability of the blend as ETL in a step towards the industrialization of PSCs. The durability of the blend under air conditions is still an open challenge beyond the scope of the paper. The technological relevance of the proposed ETL resides in the combination of the Gig-Lox sponge with a perovskite that is ideal for tandem solar cell application. ${\mathrm{CsPbI}}_{3}$ has, in fact, the desired bandgap value to satisfy the coupling with silicon solar cells, and is made more robust by the use of Europium in the formulation. As a further added value, the Gig-Lox ${\mathrm{TiO}}_{2}$ sponge was produced by sputtering, an up-scalable methodology that can be easily proposed for industrialization. The semitransparency of the ETL can also be exploited for building integrated photovoltaics. In blending the two materials, the tight interconnection at the interfaces is beneficial for an efficient injection of the photogenerated electrons from ${\mathrm{CsPbI}}_{3}$:${\mathrm{EuCl}}_{3}$ into the Gig-Lox ${\mathrm{TiO}}_{2}$ sponge that, in the end, supports its practical relevance.

## Figures and Tables

**Table 1:** X-ray penetration depths into pure ${\mathrm{CsPbI}}_{3}$ and blended material.

Grazing Angle (°)	Penetration Depth (nm) ${\mathrm{CsPbI}}_{3}$	Penetration Depth (nm) Anatase ${\mathrm{TiO}}_{2}-{\mathrm{CsPbI}}_{3}$ Blend
0.2°	3.49	3.86
0.3°	14.68	25.77
0.4°	37.28	56.84
0.6°	70.45	104.37
0.8°	98.97	145.77

**Table 2:** Crystallite size values at different scan angles using glass substrate and Gig-Lox ${\mathrm{TiO}}_{2}$ substrate.

Scan Angle (°)		FWHM (°)	Crystallite Size (nm)		FWHM (°)	Crystallite Size (nm)
0.2°	*Glass* *Substrate*	0.274	30.49	*Gig*-*Lox*$TiO_{2}$ *Substrate*	0.214	39.94
0.3°		0.312	26.56		0.271	30.85
0.4°		0.251	33.51		0.289	28.80
0.6°		0.271	30.85		0.28	29.79
0.8°		0.253	33.22		0.293	28.38

**Table 3:** Crystal parameters calculated at the correspondence Miller index. $I/I_{220}$ Exp are the reference intensities by DFT Simulation (DFT Sim). Bold values are the data reported in Figure 6.

Miller Index		Angle (°)	d (A)	Crystallite Sizes (nm)	Intensity		Angle (°)	d (A)	Crystallite Sizes (nm)	Intensity
**(002)**	*Glass* *substrate*	**14.21**	**6.228**	**40.65**	**73.7**	$TiO_{2}$ *substrate*	**14.10**	**6.275**	**24.43**	**60.1**
**(110)**		**14.41**	**6.143**	**25.81**	**139.1**		**14.33**	**6.174**	**29.99**	**117**
**(020)**		**20.66**	**4.295**	**35.50**	**242.4**		**20.63**	**4.302**	**27.20**	**139.4**
(120)		23.09	3.848	30.73	108.5		23.06	3.854	30.25	73.0
(121)		24.17	3.680	23.34	103.7		24.14	3.684	26.02	84.9
**(004)**		**28.90**	**3.087**	**50.13**	**36.3**		**28.47**	**3.132**	**21.38**	**24.6**
**(220)**		**29.08**	**3.068**	**32.51**	**178.9**		**28.97**	**3.079**	**25.28**	**96.5**
(130)		32.96	2.715	28.10	65.1		32.92	2.719	28.30	46.1
(131)		33.57	2.667	19.51	36.0		n.a.	n.a.	n.a.	n.a.
**(132)**		**36.05**	**2.490**	**29.15**	**115**		**36**	**2.493**	**26.66**	**79.3**

**Table 4:** Lattice parameters of ${\mathrm{CsPbI}}_{3}$:${\mathrm{EuCl}}_{3}$ on glass substrate and ${\mathrm{CsPbI}}_{3}$:${\mathrm{EuCl}}_{3}$ infiltrated into Gig-Lox ${\mathrm{TiO}}_{2}$ compared with reference value by Sutton et al. [36].

Lattice Parameters of the Orthorhombic γ-Phase	This Work Spin Coated Layers Quenched from 350 °C Using Eu *Glass Substrate*	This Work Spin Coated Layers Quenched from 350 °C Using Eu *Blended Material*	Sutton et al. Powder Fast Quenched from 347 °C in $N_{2}$ [36]
a [Å]	8.790	8.875	8.856
b [Å]	8.581	8.580	8.577
c [Å]	12.433	12.545	12.472
Unit cell [Å${}^{3}$]	937.78	955.27	947.33

## Data Availability

The data presented in this study are available on request from the corresponding author.

## Sample Availability

Samples are available from the authors.

## Abbreviations

The following abbreviations are used in this manuscript:

ETL	Electron-transport layer
HTL	Hole-transport layer
$TiO_{2}$	Titanium dioxide
XRD	X-ray diffraction
XRR	X-ray reflection
GIXRD	Grazing incidence X-ray diffraction
DFT	Density functional theory
PL	Photoluminescence spectroscopy
